# Morphological Differentiation Among Three Mitochondrial Lineages of *Hydrobioides nassa* Theobald, 1865 (Gastropoda: Bithyniidae) from Thailand

**DOI:** 10.3390/biology15050420

**Published:** 2026-03-04

**Authors:** Naruemon Bunchom, Bangon Kongim, Apirada Manphae, Warayutt Pilap, Ross H. Andrews, Chairat Tantrawatpan, Weerachai Saijuntha

**Affiliations:** 1 Department of Tropical Medicine and Malaria, National Institute of Global Health and Medicine, Japan Institute for Health Security, Tokyo 162-8655, Japan; aoy_narumon@hotmail.com; 2 Project for Parasitic Diseases (JICA/AMED), Vientiane 3560, Laos; 3 Department of Biology, Faculty of Science, Mahasarakham University, Maha Sarakham 44150, Thailand; kongimb@yahoo.com; 4 Scientific Instrument Academic Service Unit, Faculty of Science, Mahasarakham University, Maha Sarakham 44150, Thailand; apirada.m@msu.ac.th; 5 Walai Rukhavej Botanical Research Institute, Mahasarakham University, Maha Sarakham 44150, Thailand; warayutt@msu.ac.th; 6 Center of Excellence in Biodiversity Research, Mahasarakham University, Maha Sarakham 44150, Thailand; 7 Department of Surgery & Cancer, Faculty of Medicine, Imperial College, London SW7 2AZ, UK; rhandrews@gmail.com; 8 Division of Cell Biology, Department of Preclinical Sciences, Faculty of Medicine, and Center of Excellence in Stem Cell Research and Innovation, Thammasat University, Rangsit Campus, Pathum Thani 12120, Thailand; 9 Biomedical Science Research Unit, Faculty of Medicine, Mahasarakham University, Maha Sarakham 44000, Thailand

**Keywords:** gastropod, molecular phylogeny, shell morphology, cryptic diversity, integrative taxonomy

## Abstract

The identification of species complexes in freshwater snails remains challenging due to limited diagnostic morphological characters and incomplete taxonomic knowledge in many taxa. Within the family Bithyniidae, species have traditionally been classified using shell morphology and genital anatomy to distinguish intraspecific variation from interspecific differences. However, extensive morphological plasticity has hindered reliable species delimitation, and the presence of cryptic diversity further complicates taxonomy. Recent DNA barcoding studies of *Hydrobioides* have provided evidence of such cryptic diversity, highlighting the need for taxonomic reassessment within the genus. In the present study, we examined morphological variation in *Hydrobioides nassa* from Thailand in conjunction with mitochondrial DNA sequence data. Molecular phylogenetic analyses based on cytochrome *c* oxidase subunit I (*cox1*) sequences revealed three well-supported genetic lineages within *H. nassa*, accompanied by high levels of pairwise genetic divergence. Morphological comparisons of shell, operculum, and radular characters further supported differentiation among these lineages, although some characters showed overlap. While *Hydrobioides* has previously been regarded as comprising a single morphologically defined species, our results demonstrate that *H. nassa* represents a complex of genetically distinct lineages with subtle but consistent morphological differences. This study highlights the importance of integrating molecular approaches with traditional morphological analyses to improve taxonomic resolution and to better understand biodiversity within freshwater snail groups exhibiting cryptic diversity.

## Simple Summary

Freshwater snails are often difficult to identify because many species look very similar, even when they are genetically distinct. In this study, we examined the freshwater snail *Hydrobioides nassa* from Thailand to better understand its species boundaries. By combining DNA sequence data with detailed observations of shell and anatomical features, we found that *H. nassa* consists of three genetically distinct lineages. Although these lineages show only subtle morphological differences, these differences are consistent and support their genetic separation. Our findings demonstrate that relying on morphology alone may overlook hidden diversity and highlight the value of integrating molecular and morphological approaches to improve species identification and our understanding of freshwater snail biodiversity.

## 1. Introduction

The family Bithyniidae (Gray, 1857) is derived from the genus *Bithynia* (Leach, 1818). According to [1], the family Bithyniidae in Thailand comprises three genera: *Bithynia* (Leach, 1818) (with two subgenera, *Bithynia* (syn. *Digoniostoma* (Preston, 1910)) and *Gabbia* Tryon, 1866), *Hydrobioides* Nevill, 1884 (Brandt, 1974), and *Wattebledia* (Crosse & Fischer, 1886). Species of *Hydrobioides nassa* (Theobald, 1865) are small freshwater snails belonging to the family Bithyniidae. Freshwater snails, including this species, play important ecological roles as food resources for various aquatic organisms, such as fish and other predators [2]. In addition to their ecological importance, bithyniid snails are of considerable medical and veterinary relevance, as they may serve as intermediate hosts for food-borne parasitic diseases affecting both humans and animals in Southeast Asia, including Thailand. Several digenean trematodes have been reported to utilize bithyniid snails as first or second intermediate hosts [3,4]. Notably, *H. nassa* has been suggested as a potential intermediate host for trematode parasites, particularly lung flukes of the genus *Paragonimus* (Braun, 1899) in Nakhon Sawan Province, northern Thailand [5]. In addition, xiphidiocercariae have been detected in *H. nassa* populations from Thailand [3,4,5].

Despite their ecological and epidemiological significance, hydrobioid snails have received relatively limited scientific attention in Thailand. Most systematic studies have traditionally relied on morphological and anatomical characteristics, including shell morphology, operculum, radula, and soft tissue structures, and are often based on a limited number of specimens per species [1,6]. Although recent studies have begun to incorporate molecular genetic data and biogeographical information, such approaches remain relatively scarce [7,8]. Consequently, taxonomic research on hydrobioid snails in Thailand remains incomplete and fragmented.

Taxonomic identification within this group is particularly challenging due to pronounced morphological plasticity, whereby environmental factors can cause distinct species or genetic lineages to exhibit overlapping shell characteristics [8]. For example, *Bithynia* and *Hydrobioides* snails from northern, western, and central Thailand display similar shell morphologies despite clear genetic differentiation [4,7,8,9]. Shell morphology shows variation primarily in overall size (height and width), spiral whorls, and the body whorl, whereas the operculum remains comparatively conserved, consistently displaying a concentric pattern among individuals. These findings highlight the strong influence of ecological conditions on shell phenotypes, leading to convergent morphologies in similar habitats or divergent forms under geographic isolation [10,11]. Understanding such environmentally driven morphological variation is therefore essential for interpreting biodiversity patterns and for improving taxonomic resolution in freshwater gastropods.

In recent years, the number of newly recognized freshwater snail taxa has increased globally, largely as a result of detailed investigations of anatomical and morphological characters [12,13]. However, relatively few studies have combined traditional morphological data with molecular approaches, such as DNA barcoding, to address taxonomic complexity in this group [14]. Recently, *H. nassa* in Thailand has been classified into three genetic lineages (lineages I–III) based on molecular evidence [8], as well as bithyniid snails from the Inle Lake Basin in Myanmar, including *H. nassa*, which have been taxonomically revised using molecular genetic data, highlighting the importance of integrative approaches for resolving species boundaries [13]. Nevertheless, the taxonomy of *Hydrobioides* remains problematic due to the limited availability of comprehensive morphological and molecular datasets.

In the present study, we examined morphological variation in *H. nassa* across three genetically distinct lineages previously reported from Thailand, integrating traditional morphological analyses with molecular evidence. Our findings provide additional morphological support for lineage differentiation within *H. nassa* and contribute to filling important taxonomic gaps in the genus *Hydrobioides* in Thailand. Further studies employing integrative taxonomic approaches across broader geographic ranges are necessary to clarify the evolutionary relationships and taxonomic status of bithyniid snails throughout Southeast Asia.

## 2. Materials and Methods

### 2.1. Specimen Collection

**Figure 1:**
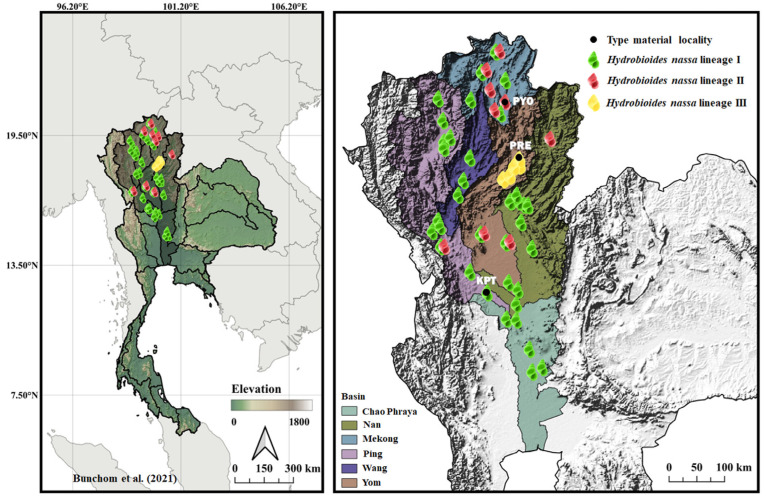
Map showing the geographical distribution of the three genetically distinct lineages (lineages I–III) of *Hydrobioides nassa* in Thailand, including the localities of the type material examined in this study [8]. PYO = Phayao, PRE = Phrae, KPT = Kamphaeng Phet.

The specimens examined in this study were ethanol-preserved samples originally collected and analyzed in a previous publication by Bunchom et al. [8]. Specimens were collected during 2016–2017 from multiple localities in northern and central Thailand. All samples were preserved in 80% ethanol and assigned codes consistent with those reported by Bunchom et al. [8]. Voucher specimens are deposited at the Center of Excellence in Biodiversity Research, Mahasarakham University, Maha Sarakham, Thailand. Based on mitochondrial cytochrome *c* oxidase subunit I (*cox1*) sequence analysis in that study, the specimens were classified into three genetically distinct lineages (lineages I–III). The four samples were collected from three habitats across multiple geographical regions in Thailand (Figure 1). Lineages were assigned to each individual based on newly generated *cox1* sequences, according to the phylogenetic criteria described by Bunchom et al. [8].

### 2.2. Morphometric Analysis

#### 2.2.1. Shell and Operculum Characteristics

Shell and operculum characteristics were examined based on shape, microsculpture, umbilicus, aperture, apex, suture, and operculum features, following the methodology [6]. Shell morphology was examined using a JEOL JSM-6460LV (JEOL Ltd., Tokyo, Japan) scanning electron microscope (SEM). Specimens were cleaned ultrasonically in tap water and dehydrated through a graded ethanol series prior to mounting on aluminum stubs with double-sided carbon adhesive tape. Samples were sputter-coated with gold to enhance conductivity before observation. Imaging was performed under high vacuum at an accelerating voltage of 10 kV and a working distance of approximately 10 mm. Secondary electron images were digitally captured for morphological analysis. Measurements were obtained from calibrated micrographs using the instrument’s image analysis software, with a precision of 0.01 mm. Shell height (H), width (W), and Aperture height (AH) were measured using a digital vernier caliper (Industrial Scientific Corporation, Pittsburgh, PA, USA).

#### 2.2.2. Radula Characteristics

Radulae were dissected and treated with a 1% sodium hydroxide (NaOH) solution, boiled for 10 min to remove soft tissues, and then rinsed several times with distilled water. The radulae were cleaned and dehydrated through a graded ethanol series prior to examination under a stereomicroscope. Residual debris was removed using fine needles and brushes. Individual radular teeth were subsequently examined and documented using scanning electron microscopy (SEM).

### 2.3. Molecular Analysis

Genomic DNA was extracted from the head-foot tissue of each specimen using the E.Z.N.A.^®^ Mollusc DNA Kit (Omega Bio-Tek Inc., Norcross, GA, USA) according to the manufacturer’s instructions. DNA quality and concentration were assessed with a NanoDrop 2000 spectrophotometer (Thermo Fisher Scientific Inc., Waltham, MA, USA). The mitochondrial *cox1* region was amplified using primers LCO1490 and HCO2198 [15]. PCR was performed in 25 µL reactions containing 18.8 µL distilled water, 2.5 µL 10× TaKaRa Ex PCR buffer (Takara Bio Inc., Kusatsu, Shiga, Japan), 0.5 µL 10 µM dNTPs, 1 µL of each primer (10 µM), 0.2 µL of TaKaRa Ex Taq DNA polymerase (5 U/µL; Takara Bio Inc., Kusatsu, Shiga, Japan), and 1 µL template DNA (~10–50 ng), following the protocol described by Bunchom et al. [8]. Thermal cycling included an initial denaturation at 94 °C for 5 min; 35 cycles of 94 °C for 30 s, 50 °C for 30 s, and 72 °C for 30 s; and a final extension at 72 °C for 8 min. Negative controls without template DNA were included in each run. PCR products (~650 bp) were separated on 1% agarose gels stained with ethidium bromide. Target bands were excised and purified using the E.Z.N.A.^®^ Gel Extraction Kit (Omega Bio-Tek Inc., Norcross, GA, USA). Purified amplicons were sequenced by 1st BASE DNA Sequencing Services (1st BASE–Apical Scientific, Seri Kembangan, Selangor, Malaysia).

### 2.4. Data Analysis

The newly generated mitochondrial *cox1* sequences (GenBank accession numbers PX506413–PX506424) were aligned together with reference sequences of freshwater snails in the family Bithyniidae retrieved from GenBank. The comparative dataset included representatives of four genera (*Bithynia*, *Gabbia*, *Wattebledia*, and *Hydrobioides*), with particular emphasis on species of the genus *Hydrobioides* (Supplementary Table S1). Sequence alignment was performed for all taxa before downstream analyses.

Phylogenetic relationships were reconstructed using both maximum likelihood (ML) and Bayesian inference (BI) approaches based on the *cox1* dataset. ML analyses were conducted under the general time-reversible model with a gamma distribution and a proportion of invariant sites (GTR + G + I) [16], with node support assessed using 1000 bootstrap replicates in MEGA v11 [17]. Bayesian inference was performed using MrBayes v3.2 [18], employing four Markov chain Monte Carlo chains run for 10 million generations, with trees sampled every 100 generations. Convergence was assessed by monitoring the average standard deviation of split frequencies, which fell below 0.01 after 73,500 sampled trees; accordingly, the first 73,500 trees were discarded as burn-in, and the remaining trees were used to construct the majority-rule consensus tree. Genetic diversity indices, including haplotype diversity (Hd) and nucleotide diversity (π), were calculated for the *cox1* dataset using DnaSP v5.10.01 [19]. Pairwise genetic distances (p-distances) were estimated using Arlequin v3.5.2.2 [20].

Comparisons of shell measurements among the three genetic groups were performed using Minitab version 18 (Minitab Inc., State College, PA, USA). Descriptive statistics, including means and standard deviations (SD), were calculated using IBM SPSS Statistics version 19 (IBM Corporation, Somers, NY, USA). Differences in trematode infection prevalence among sampling periods, locations, and lineages were analyzed using one-way analysis of variance (ANOVA). When significant differences were detected, Duncan’s Multiple Range Test [21] was applied for post hoc comparisons at a 95% confidence level.

## 3. Results

### 3.1. Molecular Phylogenetic Analyses

**Figure 2:**
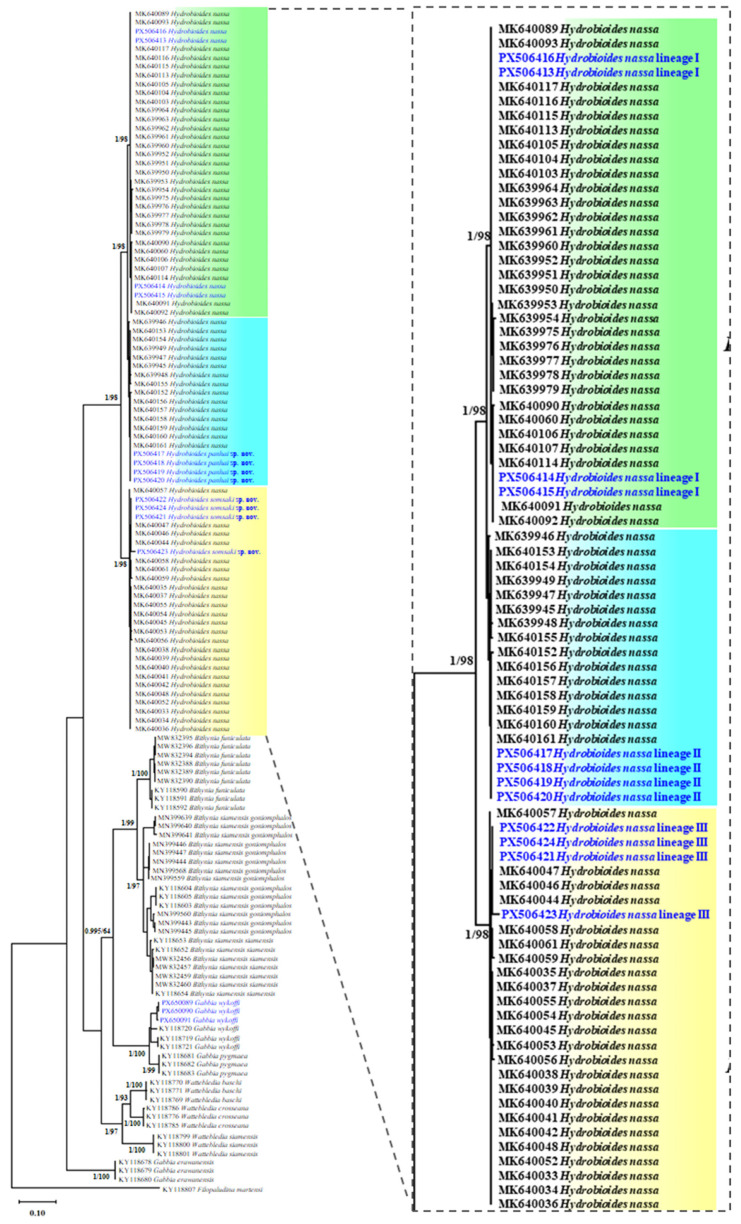
Phylogenetic tree of *Hydrobioides nassa* in Thailand based on *cox1* sequences. Taxa shown in blue were generated in this study. Nodal supports are shown as Bayesian posterior probabilities/ML bootstrap values.

All examined specimens were confirmed as members of the genus *Hydrobioides* based on mitochondrial *cox1* sequences, which showed ≥99% sequence similarity to reference sequences deposited in the GenBank database. The molecular dataset comprised *cox1* sequences (613 bp) from 12 individuals representing three lineages/localities in Thailand: lineage I from Kamphaeng Phet Province, lineage II from Phayao Province, and lineage III from Phrae Province (Figure 1). Phylogenetic analyses using BI and ML methods consistently recovered *H. nassa* as a well-supported monophyletic group (Figure 2), within which three distinct genetic lineages were resolved. Within the *cox1* dataset, 41 polymorphic sites and eight haplotypes were identified, including five unique haplotypes. Haplotype diversity (Hd) and nucleotide diversity (π) were high, with values of 0.924 ± 0.057 and 0.0275 ± 0.0042, respectively (Table 1). Pairwise *p*-distances among the three *Hydrobioides* lineages in Thailand ranged from 0.0145 to 0.0481 for the *cox1* gene (Table 2), indicating moderate to high levels of mitochondrial divergence.

### 3.2. Morphological Analyses

#### 3.2.1. Lineage I

**Figure 3:**
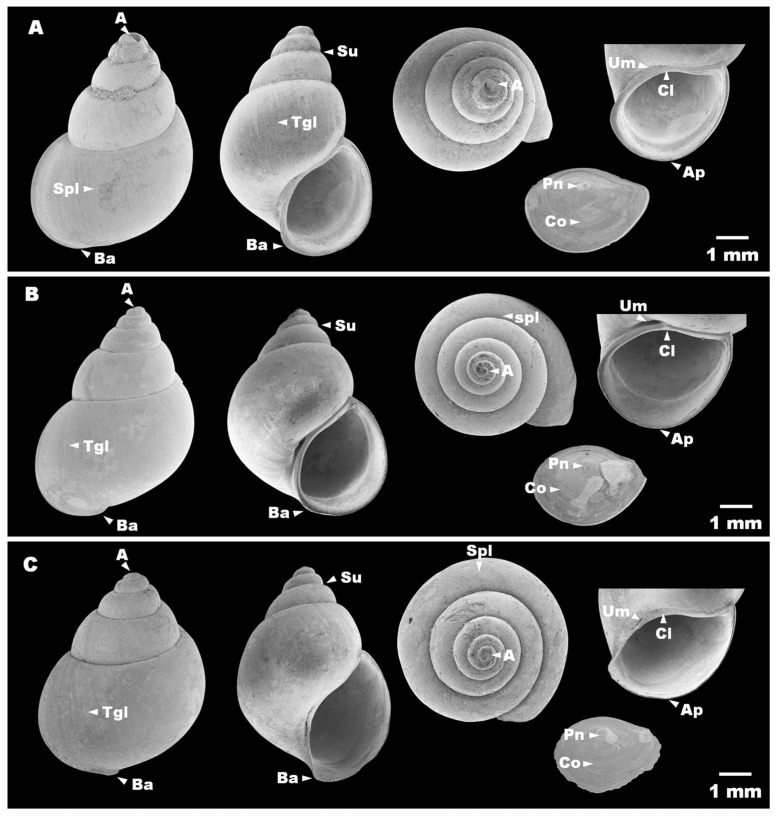
SEM images of shell and operculum characteristics of *Hydrobioides nassa*: (**A**) lineage I; (**B**) lineage II; (**C**) lineage III. A = apex, Ap = aperture, Ba = basal lip, Cl = columellar lip, Co = concentric operculum, Pn = paucispiral nucleus, Spl = spiral line, Su = suture, Tgl = transverse growth line, Um = umbilicus.

**Figure 4:**
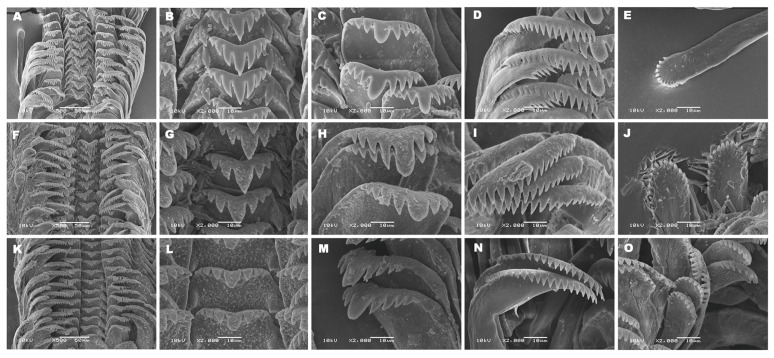
Radula characteristics of *Hydrobioides nassa*: (**A**–**E**) lineage I [(**A**) Overview of radula pattern; (**B**) Central tooth; (**C**) Lateral tooth; (**D**) Inner marginal tooth; (**E**) Outer marginal tooth], (**F**–**J**) lineage II [(**F**) Overview of radula pattern; (**G**) Central tooth; (**H**) Lateral tooth; (**I**) Inner marginal tooth; (**J**) Outer marginal tooth], (**K**–**O**) lineage III [(**K**) Overview of radula pattern; (**L**) Central tooth; (**M**) Lateral tooth; (**N**) Inner marginal tooth; (**O**) Outer marginal tooth].

Shell subovate to elongate conical (Figure 3A), consisting of five to six whorls; shell surface with fine transverse growth lines and a deep suture. Shell height 8.56 mm (range 8.01–8.83 mm) and shell width 5.27 mm (range 5.08–5.39 mm) (Figure 3A; Table 3). Surface sculpture composed of fine transverse growth lines and weak spiral striations. Umbilicus completely closed. Aperture oval, sometimes with a thickened outer lip. Operculum ellipsoid and relatively thick, with a central muscle insertion area located to the right of the nucleus and a second long, narrow attachment region extending close to the inner margin (Figure 3A). Radula taenioglossate, with the formula 2 + 1 + 1 + 1 + 2. The number of cusps on the outer marginal teeth varies among lineages, whereas the inner marginal teeth bear 17–18 cusps. Mesocones of the anterior cusps are saber-shaped (Figure 4A–E; Table 4).

**Remarks** **1.** *Shell subovate to elongate-conical, characterized by an inflated body whorl and elevated spiral whorls; a distinct varix present near the outer lip; outer lip expanded outward.*

#### 3.2.2. Lineage II

Shell broadly ovate to elongate conical (Figure 3B), consisting of six to seven whorls; shell surface with fine transverse growth lines and a deep suture. Shell height 8.55 mm (range 8.18–9.15 mm) and shell width 5.39 mm (range 4.94–5.39 mm) (Figure 3B; Table 3). Surface sculpture similar to lineage I, with fine transverse growth lines and weak spiral striations. Umbilicus relatively narrow. Aperture oval, sometimes with a thickened outer lip. Operculum ellipsoid and relatively thick, with a central muscle insertion area located to the right of the nucleus and a second long, narrow attachment region extending close to the inner margin (Figure 3B). Radula taenioglossate, with the formula 2 + 1 + 1 + 1 + 2. Inner marginal teeth bear 17–18 cusps, and the mesocones of the anterior cusps are saber-shaped (Figure 4F–J; Table 4).

**Remarks** **2.** *Shell broadly ovate to elongate-conical, characterized by elevated spiral whorls, a high aperture, and a deep suture; a distinct varix present near the outer lip; outer lip expanded outward.*

#### 3.2.3. Lineage III

Shell broadly ovate to elongate conical (Figure 3C), consisting of five to six whorls; shell surface with fine transverse growth lines and a deep suture. Shell height 8.94 mm (range 8.66–9.14 mm) and shell width 5.63 mm (range 5.51–5.70 mm) (Figure 3C; Table 3). These differences were statistically significant (*p* < 0.05). Surface sculpture composed of fine transverse growth lines and weak spiral striations. Umbilicus relatively narrow. Aperture oval, sometimes with a thinned outer lip. Operculum thinner than in lineages I and II, with a central muscle insertion area located to the right of the nucleus and a second long, narrow attachment region extending close to the inner margin (Figure 3C). Radula taenioglossate, with the formula 2 + 1 + 1 + 1 + 2. Inner marginal teeth bear 19–20 cusps, exceeding those observed in lineages I and II. Mesocones of the anterior cusps are saber-shaped (Figure 4K–O; Table 4).

**Remarks** **3.** *Shell broadly ovate to elongate-conical, characterized by a flattened body whorl and relatively low spiral whorls.*

## 4. Discussion

The morphology of several freshwater snails in the family Bithyniidae, including *H. nassa*, *Bithynia* (syn. *Digoniostoma*) *siamensis siamensis*, and *B. s. goniomphalos*, is known to be highly similar [8]. Species of *Bithynia* and *Hydrobioides* frequently coexist and are widely distributed across northern and central Thailand [4,8,9], making morphological identification and differentiation among these taxa particularly challenging [6]. These genera exhibit highly similar shell characteristics, including comparable size and a subovate to conical shell morphology, reducing the diagnostic reliability of external conchological traits alone. As a result, previous studies based solely on morphology may have led to taxonomic misidentifications, potentially biasing species distribution records in Thailand [1,6]. In this context, DNA barcoding has proven to be an effective tool for distinguishing closely related bithyniid species [4,7,8,9]. Accordingly, species identity in the present study was confirmed using mitochondrial *cox1* sequences, with all examined specimens showing ≥99% similarity to reference sequences deposited in the GenBank database and being assigned to the genus *Hydrobioides*.

Genetic diversity and population structure within *Hydrobioides* were first explored by [8], who demonstrated that *cox1* sequences clearly separate species within the family Bithyniidae, including *Hydrobioides* [7]. However, subsequent studies revealed that *cox1* alone may not fully resolve intraspecific structure in some taxa, such as *B. siamensis*, when larger sample sizes are analyzed [9]. The mitochondrial *cox1* gene is widely employed for species identification due to its conserved primer-binding regions [22], which facilitate universal amplification, coupled with sufficient sequence variability to achieve species-level discrimination. Its high copy number and comprehensive reference databases increase its utility. Nevertheless, as a maternally inherited marker, *cox1* may be influenced by hybridization or nuclear mitochondrial pseudogenes, and may provide limited resolution among recently diverged or deeply branching lineages. These findings highlight the limitations of single-locus phylogenies and emphasize the need for additional molecular markers, including nuclear genes, to improve phylogenetic resolution and taxonomic accuracy within bithyniid snails.

The number of described freshwater snail taxa continues to increase, particularly as attention has shifted toward recognizing intra- and interspecific variation. Nevertheless, the taxonomic status of many species remains uncertain due to continued reliance on shell morphology as the primary criterion for identification. Recently, a comprehensive taxonomic revision of bithyniid snails from the Inle Lake Basin in Myanmar, including *H. nassa*, incorporated molecular data and provided important insights into species boundaries within this group [13]. These studies underscore the value of integrative approaches for resolving taxonomic complexity in freshwater gastropods.

In the present study, we identified three distinct genetic lineages of *H. nassa* from Thailand based on mitochondrial phylogenetic analyses and assessed their morphological variation. *Hydrobioides nassa* lineage II exhibits a distribution overlapping with that of lineage I across northern, western, and central Thailand [8]. In contrast, lineage III appears to have a more restricted distribution, currently known only from Phrae Province in northern Thailand, and is characterized by a flatter shell and a relatively narrow umbilicus. These findings suggest that lineage III shares a common ancestral origin with lineages I and II but has undergone substantial genetic divergence over an extended evolutionary timeframe. Despite the relatively small sample size (*N* = 12), the patterns observed here are consistent with previous reports [8] and corroborated by broader regional surveys [7], supporting the widespread distribution of the three lineages across multiple provinces. Mitochondrial DNA data further indicate that lineage I is the most widespread and abundant lineage in Thailand, including outgroups [7,8,23,24]. Although mitochondrial markers such as *cox1* are valuable for species identification, reliance on a single locus has important limitations. Mitochondrial DNA reflects only maternal inheritance and may not accurately represent species boundaries due to processes such as incomplete lineage sorting, hybridization, or mitochondrial introgression. Even complete mitochondrial genomes are inherited as a single linkage unit and therefore cannot fully resolve complex evolutionary histories. Consequently, taxonomic conclusions based solely on mtDNA should be interpreted with caution and ideally supported by nuclear markers and complementary morphological evidence.

Hydrological connectivity likely plays an important role in shaping the population genetic structure of freshwater snails. Population structure is often associated with watershed systems, which can facilitate dispersal through water flow or floating vegetation. Consequently, populations inhabiting small, isolated ponds or fragmented habitats may exhibit greater genetic differentiation than geographically proximate populations connected by continuous waterways. Such landscape features may contribute to the genetic structuring observed among *H. nassa* lineages in Thailand.

This study reassessed the taxonomy of a long-recognized freshwater snail species by integrating traditional morphological analyses with DNA barcoding. While shell and radular characters provided valuable morphological information, mitochondrial sequence data revealed genetic differentiation that was not always apparent from morphology alone. The combined approach enabled a more robust evaluation of lineage boundaries within *H. nassa*, highlighting the presence of cryptic genetic diversity within morphologically similar populations. These findings reinforce the importance of integrative taxonomy for improving species delimitation and enhancing our understanding of evolutionary relationships in freshwater gastropods, with implications for biodiversity assessment and conservation planning.

Several limitations should be considered when interpreting the results of this study. Field-collected specimens included individuals at different developmental stages, and some were immature or pre-adult, which restricted the observation of certain diagnostic morphological traits. Therefore, while the inclusion of mixed developmental stages may have marginally reduced morphological resolution, it is unlikely to have introduced systematic bias into the genetic analyses or altered the overall conclusions regarding lineage differentiation. In addition, the specimens examined were ethanol-preserved samples originally collected for the study of Bunchom et al. [8]. Long-term ethanol preservation led to tissue desiccation, preventing detailed examination of soft tissues, particularly the reproductive organs. Future studies should prioritize the collection of fresh material to allow comprehensive analyses of soft anatomy across all developmental stages. Such efforts, combined with multilocus molecular data, will be essential for fully resolving the taxonomic status and evolutionary history of *H. nassa* lineages throughout Thailand and Southeast Asia.

## 5. Conclusions

The presence of three genetically distinct lineages within the *H. nassa* complex highlights the taxonomic complexity of this group and may complicate taxonomic differentiation. The integration of mitochondrial *cox1* data with morphological analyses effectively reveals cryptic diversity and improves taxonomic resolution. These findings enhance our understanding of freshwater snail biodiversity and provide a foundation for future taxonomic reassessment.

## Figures and Tables

**Table 1:** Diversity indices of the three genetic groups (lineages I–III) of *Hydrobioides nassa* from Thailand based on 613 bp *cox1* sequence analysis.

Genetic Groups	*N*	S	H	Uh	Hd ± SD	π ± SD
Lineage I	4	4	3	2	0.833 ± 0.222	0.0038 ± 0.0012
Lineage II	4	2	2	1	0.500 ± 0.265	0.0016 ± 0.0009
Lineage III	4	7	3	2	0.833 ± 0.222	0.0060 ± 0.0027
Total	12	41	8	5	0.924 ± 0.057	0.0275 ± 0.0042

*N* = number of samples, S = number of segregation sites, H = number of haplotypes, Uh = unique haplotypes, Hd = haplotype diversity, π = nucleotide diversity.

**Table 2:** Pairwise genetic distances (*p*-distance) based on *cox1* sequences among three lineages (I–III) of *Hydrobioides nassa* from Thailand.

Genetic Groups	Lineage I	Lineage II	Lineage III
Lineage I	-		
Lineage II	0.0145	-	
Lineage III	0.0465	0.0481	-

**Table 3:** Shell measurements comparison between three genetic groups (lineages I–III) of *Hydrobioides nassa* from Thailand.

Genetic Groups	*N*	H	W	AH	W/H	AH/H
Lineage I	4	8.56 ± 0.37 (8.01–8.83)	5.27 ± 0.14 ^b^ (5.08–5.39)	4.60 ± 0.03 ^b^ (4.57–4.64)	0.62 ± 0.03 (0.59–0.65)	0.54 ± 0.02 ^b^ (0.53–0.57)
Lineage II	4	8.55 ± 0.45 (8.18–9.15)	5.25 ± 0.21 ^b^ (4.94–5.39)	4.89 ± 0.18 ^a^ (4.67–5.06)	0.61 ± 0.03 (0.58–0.65)	0.57 ± 0.01 ^a^ (0.55–0.59)
Lineage III	4	8.94 ± 0.21 (8.66–9.14)	5.63 ± 0.08 ^a^ (5.51–5.70)	4.45 ± 0.09 ^b^ (4.32–4.53)	0.63 ± 0.01 (0.62–0.64)	0.50 ± 0.00 ^c^ (0.49–0.50)

Different superscript letters (a, b, c) within the same column indicate statistically significant differences (*p* < 0.05). *N* = number of samples, H = height, W = width, AH = aperture height, W/H = width/height, AH/H = aperture height/height.

**Table 4:** Comparative shell, opercular, and radular morphological characters of *Hydrobioides nassa* and its three genetic lineages from Thailand.

Sample	Shell Shape	Size (mm)		Spire Angle	Number of Whorls	Umbilicus	Operculum	Aperture	Radula Characteristics			References
		H	W						LFT (Cusps)	IMT (Cusps)	OMT (Cusps)	
*H. nassa*	Ovately conic	6.20–11.80	4.40–7.00	-	-	Completely closed	Concentric	Large, ovate, bluish-white	(2-4)-1-(3-5)	15–18	17–22	[1]
*H. nassa*	Subovately conic	9.65	5.58	52.98°	5 1/2–6 1/4	Umbilical chink	Concentric	Angulate at the left aperture with a varix side of the base	(3-4)-1-(3-4)	19–21	10–13	[6]
Lineage I	Subovately conic	8.56 (8.01–8.83)	5.27 (5.08–5.39)	45.00° (44–46)	5 1/6	Completely closed	Concentric	Angulated at the left aperture with a slightly curved basal lip	(3-4)-1-(3-4)	17–18	13–14	This study
Lineage II	Broadly (ovately) conic	8.55 (8.18–9.15)	5.25 (4.94–5.39)	54.25° (53–55)	6 1/6	Relatively narrow	Concentric	Unsharply angulated at the left aperture with a rounded basal lip	(3-4)-1-(3-4)	17–18	15–18	This study
Lineage III	Broadly (ovately) conic	8.94 (8.66–9.14)	5.63 (5.51–5.70)	59.00° (58–60)	6	Relatively narrow	Concentric	Sharply angulated at the left aperture with a varix side of the base	(3-4)-1-(3-4)	19–20	10–12	This study

H = height, W = width, LFT = lateral tooth formula, IMT = inner marginal tooth, OMT = outer marginal tooth.

## Data Availability

All data are available upon request.

## Supplementary Materials

The following supporting information can be downloaded at: https://www.mdpi.com/article/10.3390/biology15050420/s1, Table S1: Voucher information and GenBank accession numbers for *Hydrobioides nassa* from Thailand included in the present study.
