# Supplementary material for: Morphological Differentiation Among Three Mitochondrial Lineages of Hydrobioides nassa Theobald, 1865 (Gastropoda: Bithyniidae) from Thailand
Source: Biology (Basel). 2026 Mar 4;15(5):420. doi: 10.3390/biology15050420 (PMC12984682; doi:10.3390/biology15050420)
Supplement: Supplementary file 1 [file biology-15-00420-s001.zip › Supplementary Materials_27022026.pdf]

Supplementary: Table S1. Voucher information and Genbank accession numbers for freshwater snails in the genus *Hydrobioides nassa* from Thailand included in the present study.

Species	Isolation	Haplogroup			Genbank accession numbers	References
		**I**	**II**	**III**	***cox1***	
Hydrobioides nassa lineage I	HnKPT1	/	-	-	PX506413	This study
H. nassa lineage I	HnKPT2	/	-	-	PX506414	This study
H. nassa lineage I	HnKPT3	/	-	-	PX506415	This study
H. nassa lineage I	HnKPT4	/	-	-	PX506416	This study
H. nassa	HnC1401	/	-	-	MK639950	[8]
H. nassa	HnC1402	/	-	-	MK639951	[8]
H. nassa	HnC1403	/	-	-	MK639952	[8]
H. nassa	HnC1404	/	-	-	MK639953	[8]
H. nassa	HnC1405	/	-	-	MK639954	[8]
H. nassa	HnC1601	/	-	-	MK639960	[8]
H. nassa	HnC1602	/	-	-	MK639961	[8]
H. nassa	HnC1603	/	-	-	MK639962	[8]
H. nassa	HnC1604	/	-	-	MK639963	[8]
H. nassa	HnC1605	/	-	-	MK639964	[8]
H. nassa	HnC1801	/	-	-	MK639975	[8]
H. nassa	HnC1802	/	-	-	MK639976	[8]
H. nassa	HnC1803	/	-	-	MK639977	[8]
H. nassa	HnC1804	/	-	-	MK639978	[8]

H. nassa	HnC1805	/	-	-	MK639979	[8]
H. nassa	HnC3304	/	-	-	MK640060	[8]
H. nassa	HnC3401	/	-	-	MK640103	[8]
H. nassa	HnC3402	/	-	-	MK640104	[8]
H. nassa	HnC3403	/	-	-	MK640105	[8]
H. nassa	HnC3404	/	-	-	MK640106	[8]
H. nassa	HnC3405	/	-	-	MK640107	[8]
H. nassa	HnC3701	/	-	-	MK640113	[8]
H. nassa	HnC3702	/	-	-	MK640114	[8]
H. nassa	HnC3703	/	-	-	MK640115	[8]
H. nassa	HnC3704	/	-	-	MK640116	[8]
H. nassa	HnC3705	/	-	-	MK640117	[8]
H. nassa	HnS1801	/	-	-	MK640089	[8]
H. nassa	HnS1802	/	-	-	MK640090	[8]
H. nassa	HnS1803	/	-	-	MK640091	[8]
H. nassa	HnS1804	/	-	-	MK640092	[8]
H. nassa	HnS1805	/	-	-	MK640093	[8]
H. nassa lineage II	HnPYO1	-	/	-	PX506417	This study
H. nassa lineage II	HnPYO2	-	/	-	PX506418	This study
H. nassa lineage II	HnPYO3	-	/	-	PX506419	This study
H. nassa lineage II	HnPYO4	-	/	-	PX506420	This study
H. nassa	HnC2101	-	/	-	MK639945	[8]
H. nassa	HnC2102	-	/	-	MK639946	[8]

H. nassa	HnC2103	-	/	-	MK639947	[8]
H. nassa	HnC2104	-	/	-	MK639948	[8]
H. nassa	HnC2105	-	/	-	MK639949	[8]
H. nassa	HnC2301	-	/	-	MK640152	[8]
H. nassa	HnC2302	-	/	-	MK640153	[8]
H. nassa	HnC2303	-	/	-	MK640154	[8]
H. nassa	HnC2304	-	/	-	MK640155	[8]
H. nassa	HnC2305	-	/	-	MK640156	[8]
H. nassa	HnC2401	-	/	-	MK640157	[8]
H. nassa	HnC2402	-	/	-	MK640158	[8]
H. nassa	HnC2403	-	/	-	MK640159	[8]
H. nassa	HnC2404	-	/	-	MK640160	[8]
H. nassa	HnC2405	-	/	-	MK640161	[8]
H. nassa lineage III	HnPRE1	-	-	/	PX506421	This study
H. nassa lineage III	HnPRE2	-	-	/	PX506422	This study
H. nassa lineage III	HnPRE3	-	-	/	PX506423	This study
H. nassa lineage III	HnPRE4	-	-	/	PX506424	This study
H. nassa	HnC3001	-	-	/	MK640038	[8]
H. nassa	HnC3002	-	-	/	MK640039	[8]
H. nassa	HnC3003	-	-	/	MK640040	[8]
H. nassa	HnC3004	-	-	/	MK640041	[8]
H. nassa	HnC3005	-	-	/	MK640042	[8]
H. nassa	HnC3101	-	-	/	MK640044	[8]

H. nassa	HnC3102	-	-	/	MK640045	[8]
H. nassa	HnC3103	-	-	/	MK640046	[8]
H. nassa	HnC3104	-	-	/	MK640047	[8]
H. nassa	HnC3105	-	-	/	MK640048	[8]
H. nassa	HnC3201	-	-	/	MK640052	[8]
H. nassa	HnC3202	-	-	/	MK640053	[8]
H. nassa	HnC3203	-	-	/	MK640054	[8]
H. nassa	HnC3204	-	-	/	MK640055	[8]
H. nassa	HnC3205	-	-	/	MK640056	[8]
H. nassa	HnC3301	-	-	/	MK640057	[8]
H. nassa	HnC3302	-	-	/	MK640058	[8]
H. nassa	HnC3303	-	-	/	MK640059	[8]
H. nassa	HnC3305	-	-	/	MK640061	[8]
H. nassa	HnS1701	-	-	/	MK640033	[8]
H. nassa	HnS1702	-	-	/	MK640034	[8]
H. nassa	HnS1703	-	-	/	MK640035	[8]
H. nassa	HnS1704	-	-	/	MK640036	[8]
H. nassa	HnS1705	-	-	/	MK640037	[8]
Bithynia funiculata	BfC1501	-	-	-	MW832394	[8]
B. funiculata	BfC1502	-	-	-	MW832395	[8]
B. funiculata	BfC1503	-	-	-	MW832396	[8]
B. funiculata	BfC1801	-	-	-	MW832388	[8]

B. funiculata	BfC1802	-	-	-	MW832389	[8]
B. funiculata	BfC1803	-	-	-	MW832390	[8]
B. funiculata	JUT087-12	-	-	-	KY118590	[7]
B. funiculata	JUT085-12	-	-	-	KY118591	[7]
B. funiculata	UT078-12	-	-	-	KY118592	[7]
B. siamensis goniomphalos	BsgAm1	-	-	-	MN399559	[23]
B. s.goniomphalos	BsgAM2	-	-	-	MN399560	[23]
B. s.goniomphalos	BsgAm10	-	-	-	MN399568	[23]
B. s.goniomphalos	BsgCKc1	-	-	-	MN399639	[23]
B. s.goniomphalos	BsgCKc2	-	-	-	MN399640	[23]
B. s.goniomphalos	BsgCKc3	-	-	-	MN399641	[23]
B. s.goniomphalos	BsgMDm1	-	-	-	MN399443	[23]
B. s.goniomphalos	BsgMDm2	-	-	-	MN399444	[23]
B. s.goniomphalos	BsgMDm3	-	-	-	MN399445	[23]
B. s.goniomphalos	BsgMDm4	-	-	-	MN399446	[23]
B. s.goniomphalos	BsgMDm5	-	-	-	MN399447	[23]
B. s.goniomphalos	JUT059-12	-	-	-	KY118603	[7]
B. s.goniomphalos	JUT058-12	-	-	-	KY118604	[7]
B. s.goniomphalos	JUT057-12	-	-	-	KY118605	[7]
B. s. siamensis	BssS0101	-	-	-	MW832456	[8]
B. s. siamensis	BssS0102	-	-	-	MW832457	[8]
B. s. siamensis	BssS0201	-	-	-	MW832459	[8]

B. s. siamensis	BssS0202	-	-	-	MW832460	[8]
B. s. siamensis	JUT024-12	-	-	-	KY118652	[7]
B. s. siamensis	JUT023-12	-	-	-	KY118653	[7]
B. s. siamensis	JUT022-12	-	-	-	KY118654	[7]
Gabbia erawanensis	JUT091-12	-	-	-	KY118678	[7]
Gabbia erawanensis	JUT092-12	-	-	-	KY118679	[7]
Gabbia erawanensis	JUT093-12	-	-	-	KY118680	[7]
G. pygmaea	JUT096-12	-	-	-	KY118681	[7]
G. pygmaea	JUT097-12	-	-	-	KY118682	[7]
G. pygmaea	JUT095-12	-	-	-	KY118683	[7]
G. wykoffi	JUT152-12	-	-	-	KY118719	[7]
G. wykoffi	JUT151-12	-	-	-	KY118720	[7]
G. wykoffi	JUT150-12	-	-	-	KY118721	[7]
G. wykoffi	Gw_A11-1	-	-	-	PX650089	This study
G. wykoffi	Gw_A11-2	-	-	-	PX650090	This study
G. wykoffi	Gw_A11-3	-	-	-	PX650091	This study
Wattebledia baschi	JUT130-12	-	-	-	KY118769	[7]
W. baschi	JUT129-12	-	-	-	KY118770	[7]
W. baschi	JUT128-12	-	-	-	KY118771	[7]
W. crosseana	JUT178-12	-	-	-	KY118776	[7]
W. crosseana	JUT203-12	-	-	-	KY118785	[7]
W. crosseana	JUT202-12	-	-	-	KY118786	[7]

W. siamensis	JUT106-12	-	-	-	KY118799	[7]
W. siamensis	JUT239-12	-	-	-	KY118800	[7]
W. siamensis	JUT103-12	-	-	-	KY118801	[7]
Filopaludina martensi	JUT219-12	-	-	-	KY118807	[7]
F. bengalensis	Bb-Od_1	-	-	-	-	[24]
